# Health‐promoting bioactivity and in vivo genotoxicity evaluation of a hemiepiphyte fig, *Ficus dubia*

**DOI:** 10.1002/fsn3.2205

**Published:** 2021-03-03

**Authors:** Uthaiwan Suttisansanee, Pornsiri Pitchakarn, Pisamai Ting, Woorawee Inthachat, Parunya Thiyajai, Daraphan Rodthayoy, Jirarat Karinchai, Bhanumas Chantarasuwan, Onanong Nuchuchua, Piya Temviriyanukul

**Affiliations:** ^1^ Institute of Nutrition Mahidol University Salaya, Phuttamonthon Nakhon Pathom Thailand; ^2^ Food and Nutrition Academic and Research Cluster Institute of Nutrition Mahidol University Salaya, Phuttamonthon Nakhon Pathom Thailand; ^3^ Department of Biochemistry Faculty of Medicine Chiang Mai University Meung Chiang Mai Thailand; ^4^ Thailand Natural History Museum National Science Museum Pathum Thani Thailand; ^5^ National Nanotechnology Center (NANOTEC) National Science and Technology Development Agency (NSTDA) Pathum Thani Thailand

**Keywords:** antidiabetes, antioxidant, cancer, *Drosophila melanogaster*, *Ficus dubia*, *Ficus* species

## Abstract

*Ficus* species have been used as a typical component in food and folk medicine in Asia for centuries. However, little is known regarding the bioactivity and genotoxicity of the recently identified *Ficus dubia* (FD), an indigenous plant of the tropical evergreen rain forest. FD is unique from other *Ficus* species because of its highly sought‐after red‐brown latex. Antioxidant properties together with phenolic and flavonoid contents of FD were elucidated. Health‐promoting characteristics were examined by studying the inhibition of enzymes as a drug target for diabetes, hypertension, Alzheimer's disease, and obesity, together with anticancer ability against human colorectal adenocarcinoma, human hepatocellular carcinoma, human ovarian carcinoma, human prostate adenocarcinoma, and human lung carcinoma. Besides, FD genotoxicity was tested using the *Drosophila* wing spot test. Results showed that both FD root and latex exhibited antioxidant activity due to the presence of phenolics and flavonoids, specifically caffeic acid and cyanidin. The ethanolic fraction of FD root demonstrated a potent antidiabetic mechanism underlying α‐glucosidase inhibitory activity similar to acarbose. This fraction also suppressed lung and ovarian cancer growth, possibly by G1 and G2/M arrest, respectively. All tested fractions lacked mutagenicity in vivo. Results indicated that FD can be developed as novel antidiabetic compounds; however, its bioactive compounds should be further identified.

## Graphical abstract

explored phenolic profile, health‐promoting activity, and genotoxicity assessment of *Ficus dubia* (FD), an indigenous plant of the tropical evergreen rain forest. Results indicated that FD can be developed as novel antidiabetic and anticancer compounds.

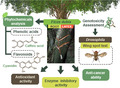

## 1 INTRODUCTION

Noncommunicable diseases (NCDs) and neurodegenerative diseases (NRDs) are becoming a significant risk factor for premature mortality and morbidity in humans. NCDs are allied with four disease groups as cardiovascular diseases, chronic pulmonary diseases, cancers, and diabetes (Hunter & Reddy, 2013). In 2016, NCDs resulted in 72.3% or 39.5 million deaths globally (The Lancet, 2017), with 80% from low‐income and middle‐income countries including Thailand. Neurodegenerative diseases are age‐dependent disorders, and now commonly found because of the increased life expectancy in the elderly. Neurodegenerative diseases such as Alzheimer's, Parkinson's, and amyotrophic lateral sclerosis (ALS) are characterized by neuronal cell death within the brain resulting in dementia, cognitive impairment, reduced ability in daily living, and death. To date, a diverse range of medicines have been used to treat NCDs and NRDs; however, several drugs have been reported to have severe side effects and poor efficacy. Thus, the quest for natural substances having potential to treat NCDs or NRDs with minimal or no side effects is warranted.

Fig trees comprise the genus *Ficus* in the family Moraceae, one of the most abundant plant genera with around 800 species worldwide. Some 500 *Ficus* species have been recorded in Asian‐Australasian tropical region, with growth habits varying from free‐standing woody trees to small shrubs, vines, lithophytes, epiphytes, hemiepiphytes, and rheophytes (Rønsted et al., 2008; Woodland, 2009). Leaves, bark, roots, latex, and fruits of *Ficus* spp. have all been utilized as folk medicine in Thai and Chinese cultures and Ayurveda for centuries (Lansky et al., 2008; Nutmakul et al., 2016) and consumed as food. The fruits of *Ficus carica* (*F. carica*), the common fig, are traded globally. Fresh or dried fruits of the common fig have a good taste and are high in minerals, vitamins, fiber, and phytonutrients including rutin, chlorogenic acid, triterpenoids, anthocyanins, and flavan‐3‐ol monomers (Wojdyło et al., 2016). Other *Ficus* species have also been used as medicine for a wide range of ailments covering the endocrine system, gastrointestinal tract, central nervous system, inflammation, and cancer. An ethanolic extract of *Ficus racemosa* inner bark, stem milky sap, and frozen fruit, and stem of *Ficus tikoua* inhibited cyclooxygenase‐1 (COX‐1) activity, resulting in poor biosynthesis of inflammatory mediators, prostaglandin E2 (PGE_2_), and prostaglandin D2 (PGD_2_) (Li et al., 2003). Isolated 6‐O‐acyl‐β‐D‐glucosyl‐β‐sitosterols (6‐AGS) from *F. carica* latex showed anticancer activities against many cancer cells such as Burkitt B‐cell lymphoma (DG‐75 cells) and T‐cell leukemia (Jurkat cells) (Rubnov et al., 2001). The fruit of *F. carica* and bark of *F. benghalensis* have been reported for their potent antidiabetic properties by inhibiting α‐glucosidase enzyme in vitro (Ahmed et al., 2011; Wojdyło et al., 2016), leading to reduced glucose absorption in the intestinal tract. *Ficus* spp. contain numerous health‐promoting substances including flavonoids, alkaloids, triterpenoids, tannins, β‐sitosterols, and phenolic acids (Lansky et al., 2008). These phytochemicals have been intensively studied for their aptitude to prevent or treat diseases, particularly as anticancer therapy and illness related to metabolic syndromes (Hu et al., 2012; Shahidi & Ambigaipalan, 2015).

*Ficus dubia* (FD), a hemiepiphytic *Ficus*, is an indigenous plant that grows in the tropical evergreen rain forests of Southern Thailand, Malaysia, and Brunei (Chantarasuwan & Thong‐Aree, 2006). The tree can reach up to 30–35 m with auxiliary aerial roots lengthening to the ground from branches or the trunk. The latex within the wood has been used to prevent infection or healing. Intriguingly, the unique characteristic of FD is its red‐brown latex after exposure to the air. Local people believe that this striking latex color may offer health benefits through anticancer and antidiabetic properties. Although there is no scientific information supporting its ethnopharmacological properties and safety, the price of FD latex is around 100–200 US dollars/kg. This is the first report to investigate the bioactivity, health benefits, and genotoxicity of FD root and latex. Antioxidant properties were determined, together with phenolic acid and flavonoid profiles of FD root and latex. Health benefits were studied using inhibitory activities against crucial enzymes involved in NCDs and NRDs including α‐glucosidase, angiotensin‐converting enzyme, acetylcholinesterase, and lipase as drug targets for diabetes, hypertension, Alzheimer's disease, and obesity, respectively. Anticancer activities against five human cancer cell lines as colorectal adenocarcinoma (SW620), hepatocellular carcinoma (HepG2), ovarian carcinoma (SKOV‐3), prostate adenocarcinoma (PC3), and lung carcinoma (A549) were also investigated. A genotoxicity assessment of FD was also performed using the in vivo wing spot test (SMART) in *Drosophila*.

## 2 MATERIALS AND METHODS

### 2.1 Plant material and collection

Roots and latex of *F. dubia* were collected from Narathiwat Province, the Southern province of Thailand. The plant was collected and identified by Dr. Bhanumas Chanatarasuwan (Taxonomist), and a voucher specimen (Chantarasuwan 040117‐1) was deposited at Thailand Natural History Museum, National Science Museum, Thailand.

### 2.2 Sample preparation and extraction

#### 2.2.1 Root preparation and extraction

*Ficus dubia* roots were prepared according to (Jin et al., 2015) with slight modification. In brief, roots were washed with tap water twice, followed by deionized water. They were then cut into small pieces with a thickness of 1.5–2 cm and dried at 40°C for ten days. The dried samples were finely powdered with an electric mill and kept in a desiccator until required for analysis.

*Normal extraction*: Ten grams of finely powdered roots were mixed with 100 ml of deionized water (RW30) or 80% ethanol (REt30). Then, mixtures were shaken at 30°C for 12 hr. The supernatants were collected by centrifugation at 939 *g* for 10 min, and the residues were subsequently re‐extracted using the same solvent and temperature. A rotatory evaporator was employed to remove the solvent.

*Hot extraction*: The sample was extracted following the same method as normal extraction, except that only deionized water was used, and the extraction temperature was set at 80°C (RW80).

#### 2.2.2 Latex preparation and extraction

*Ficus dubia* latex was freeze‐dried. Then, dried latex powder was redissolved in deionized water at 1 mg/ml. The solution was filtered through Whatman^®^ No.1 paper several times to remove any latex residuals and then freeze‐dried again. The dried latex sample was stored at −20°C until required for analysis.

### 2.3 Phytochemical analysis

The Folin–Ciocalteu assay was used to measure total phenolic contents (TPCs) as formerly detailed (Thuphairo et al., 2019). Gallic acid varying from 10 to 200 µg/ml was used as a reference compound and total phenolic contents were displayed as mg gallic acid equivalent (GAE)/g extract. Total flavonoid contents (TFCs) were determined by the aluminum chloride method using quercetin as a reference compound (Thuphairo et al., 2019) and expressed as mg quercetin equivalent (QE)/g extract. High‐performance liquid chromatography (HPLC) was used for identification and quantification against authentic standards (Charoenkiatkul et al., 2016) including phenolic acids (gallic acid, 4‐hydroxybenzolic acid, vanillic acid, syringic acid, caffeic acid, p‐coumaric acid, ferulic acid, and sinapic acid) and flavonoids (luteolin, apigenin, naringenin, hesperitin, myricetin, quercetin, kaempferol, isorhamnetin, delphinidin, cyanindin, peonidin, pelargonidin, and petunidin).

### 2.4 Antioxidant estimation

Antioxidant activity was measured by several methods including 2,2‐diphenyl‐1‐picrylhydrazyl (DPPH) scavenging activity, ferric ion reducing antioxidant power (FRAP), oxygen radical absorbance capacity (ORAC), and oxygen radical absorbance capacity (ABTS) (Benzie & Strain, 1996; Biskup et al., 2013; Fukumoto & Mazza, 2000; Prior et al., 2003; Temviriyanukul et al., 2020).

### 2.5 Enzyme inhibitory assay

Enzyme inhibitory assay against α‐glucosidase, acetylcholinesterase (AChE), angiotensin‐converting enzyme (ACE), and lipase was determined using well‐established protocols (Choi et al., 2003; Jimsheena & Gowda, 2009; Jung et al., 2009; Oboh et al., 2011; Thuphairo et al., 2019).

### 2.6 Cytotoxicity analysis

The MTT assay was used to determine cellular toxicity against normal cells, or human cancer cell lines as formerly described (Pitchakarn et al., 2017). Colorectal adenocarcinoma (SW620), hepatocellular carcinoma (HepG2), ovarian carcinoma (SKOV‐3), prostate adenocarcinoma (PC3), and lung carcinoma (A549) were used and all cell lines were purchased from the American Type Culture Collection (ATCC). All cancer cells were exposed to various concentrations of RW30, REt30, RW80, and latex for 48 hr at 37°C in a 5% CO_2_ humidified atmosphere before analysis. At the indicated time, MTT dye was added. Formazan production was determined by spectrophotometry at 540/630 nm. To determine the selectivity index (SI) of REt30, mouse embryonic fibroblast (3T3‐L1) was used to represent normal cells. The SI index was calculated as the IC_50_ ratio of REt30 on 3T3‐L1 to each cancer cell line at the same duration time of treatment. An SI value ≥2 indicated that the cytotoxic REt30 was selective against cancer cells (Badisa et al., 2006).

### 2.7 Cell cycle analysis by flow cytometry

A549 and SKOV3 cells were treated with increasing concentrations of REt30 for 48 hr, and then, cell suspensions were prepared and stained with propidium iodide (Guava^®^ cell cycle reagent; Guava Technologies) according to the Guava^®^ Cell Cycle Assay protocol. Cell cycle phase distributions were determined on a Guava^®^ PCA Instrument using CytoSoft Software. Cisplatin at IC_50_ (14 and 10 µM for A549 and SKOV3, respectively) was used as a positive control.

### 2.8 In vivo genotoxicity analysis in *Drosophila*

To determine the mutagenic potential of RW30, REt30, RW80, and latex, the somatic mutation and recombination test (SMART) were employed (de Andrade et al., 2004). One hundred 3‐day‐old trans‐heterozygous larvae carrying mwh flr^+^/mwh TM3 were fed with normal *Drosophila* medium (NDM) containing (a) deionized water (negative control), (b) 20 mM urethane (positive control), (c) RW30, (d) REt30, (e) RW80, and (f) latex. Five days after the first hatching, the number of flies was counted to determine the survival rate. Wings from flies expressing round wings were cut and mounted with Faure's solution. The phenotypes and statistical analysis were performed as previously explained (Frei & Wurgler, 1988; Graf et al., 1984).

## 3 RESULTS AND DISCUSSION

### 3.1 Antioxidant activity

Oxidants contribute to degenerative diseases including diabetes, cancer, and premature aging (Gupta et al., 2014). Enhancing the antioxidant capacity in the body can prevent these clinical disorders. *Ficus dubia* was first determined for its antioxidant activity through the two mechanisms of hydrogen atom transfer (HAT) and single electron transfer (SET). The HAT mechanism can be measured by ABTS and ORAC assays, while the SET reaction can be measured by DPPH and FRAP assays (Liang & Kitts, 2014). As shown in Table 1, among all extracts, REt30 exhibited the highest antioxidant activities determined by DPPH and FRAP assays, indicating oxidant reduction via SET reaction. Ethanol reduced the polarity index and enhanced extraction conditions of FD root by enriching bioactive compounds. A previous study found that the SC_50_ value determined by DPPH radical scavenging assay of methanolic extracted *F. benjamina* root grown in Pakistan was 58.81 ± 4.50 μg/ml or 4.3‐fold greater than REt30 (Imran et al., 2014), indicating that species, growth location, and extraction methods all contributed to the result. A contradiction was shown between ABTS and ORAC of latex (Table 1); however, FD latex quenched oxygen radicals better than the root (3‐ to 5‐fold). The HAT reaction mechanism may be responsible for reducing oxygen radicals. The ORAC assay was proven to be more relevant to the body as it uses a biological radical source (Prior et al., 2003).

**TABLE 1:** Antioxidant activities of RW30, RW80, REt30, and latex

Sample	ABTS radical scavenging activity SC_50_ value (µg/ml)	DPPH radical scavenging activity SC_50_ value (µg/ml)	FRAP (µmol TE/g extract)	ORAC (µmol TE/g extract)
RW30	56.51 ± 2.24^B^	611.30 ± 36.92^A^	515 ± 37.90^C^	1,678 ± 94^D^
RW80	38.76 ± 3.26^D^	460.55 ± 18.08^C^	601 ± 21.10^B^	2,059 ± 107^C^
REt30	43.39 ± 0.38^C^	250.31 ± 102.66^D^	830 ± 22.75^A^	2,671 ± 85^B^
Latex	87.09 ± 0.89^A^	579.67 ± 15.03^B^	461 ± 34.67^D^	7,976 ± 70^A^
Catechin	2.04 ± 0.03^E^	4.42 ± 0.06^E^	ND	ND

Notes

Data are presented as mean ± *SD* of triplicate determinations. Capital letter within a column for a given parameter is significantly different from each other at *p* <.05. The statistical package for social sciences (SPSS) was used to calculate for statistical difference by one‐way analysis of variance (ANOVA) and Duncan's multiple comparison test.

Abbreviation: ND, not determined.

### 3.2 Phytochemical analysis

The presence of phytochemicals such as phenolic and flavonoid compounds may contribute to antioxidant capacity (Pandey & Rizvi, 2009). Therefore, we further investigated total phenolic (TPCs) and total flavonoid contents (TFCs) of FD. Results in Table 2 show that FD root was rich in both phenolic and flavonoid compounds, especially in REt30. These results were consistent, suggesting that high antioxidant activity of REt30 following DPPH and FRAP may associate with high amounts of TPCs and TFCs (Tables 1 and 2) (Imran et al., 2014). Remarkably, the latex fraction showed the highest amount of TPCs at 1‐ to 2‐fold higher than the root extract, whereas TFC level was the lowest at 3‐ and 4‐fold lower than root extracts, implying that oxygen radical absorbance capacity (ORAC) in latex may primarily result from phenolic compounds. Furthermore, TPCs and TFCs of *F. religiosa* latex were reported as 2.76 ± 0.84 mg GAE/g extract and 1.84 mg QE/g extract, respectively (Yadav, 2015). We then hypothesized that the red‐brown pigments presented in FD latex might belong to groups of phenolic and flavonoid compounds. Moreover, considering the TFCs in RW80, some flavonoids presented in the root may be heat‐labile types.

**TABLE 2:** Total phenolic contents (TPCs) and total flavonoid contents (TFCs) of RW30, RW80, REt30, and latex

Sample	Total phenolic contents (mg GAE/g extract)	Total flavonoid contents (mg QE/g extract)
RW30	137.85 ± 5.75^C^	319.12 ± 17.16^B^
RW80	133.71 ± 1.11^C^	269.78 ± 16.68^C^
REt30	249.20 ± 3.72^B^	359.87 ± 5.79^A^
Latex	311.01 ± 13.34^A^	88.93 ± 6.52^D^

Notes

Data are presented as mean ± *SD* of triplicate experiments. Capital letter within a column for a given parameter is significantly different from each other at *p* <.05. The SPSS program was used to calculate for statistical difference by one‐way analysis of variance (ANOVA) and Duncan's multiple comparison test.

HPLC analysis was applied for further investigation of phenolic acids and flavonoids in FD using twenty‐one phenolic and flavonoid standards. First, undigested FD extracts were subjected for analysis; however, no phenolic acids or flavonoids were identified (data not shown). Thereafter, prior to HPLC analysis, all FD extracts were subjected to acid hydrolysis to remove sugar groups. The chromatograms are shown in Figure S1. From these chromatograms, only two compounds were identified and quantified. FD root contained both caffeic acid and cyanidin as phenolic acid and flavonoid, respectively (Table 3). The REt30 fraction had the highest amount of caffeic acid and cyanidin that correlated with these results (Table 2). Intriguingly, *F. benjamina* root contained chlorogenic acid, syringic acid, p‐coumaric acid, and ferulic acid but not caffeic acid (Imran et al., 2014). Table 3 also demonstrates that latex contained caffeic acid but not cyanidin. Anthocyanins, as members of the flavonoid group, contribute to red and purple colors in fruits. Our data indicated that the red‐brown color of FD latex may not originate from cyanidin.

**TABLE 3:** Phenolic acid and flavonoid identification of RW30, RW80, REt30, and latex after acid hydrolysis by HPLC compared with authentic standards

Sample	Phenolic acid: Caffeic acid (µg/100 g extract)	Flavonoid: Cyanidin (µg/100 g extract)
RW30	272.90 ± 0.97^D^	7.49 ± 2.97^C^
RW80	1,452.14 ± 26.51^C^	94.03 ± 13.39^B^
REt30	2,720.86 ± 186.03^A^	449.39 ± 17.45^A^
Latex	1,559.85 ± 15.10^B^	ND

Notes

Data are presented as mean ± *SD* of triplicate experiments. Capital letter within a column for a given parameter are significantly different from each other at *p* <.05. The SPSS program was used to calculate for statistical difference by one‐way analysis of variance (ANOVA) and Duncan's multiple comparison test.

ND, not detected.

### 3.3 Enzyme inhibitory properties

*Ficus* species have been recognized for their value as rich sources of phytochemicals with therapeutic potential. To confirm this, FD extracts were tested for their beneficial health properties against key enzymes involved in NCDs and NRDs including α‐glucosidase, ACE, AChE, and lipase as drug targets for diabetes, hypertension, Alzheimer's disease, and obesity, respectively. Table 4 shows the enzyme inhibitory activities of FD extracts. Results indicated that REt30 exhibited higher inhibitory effect against both α‐glucosidase and ACE at IC_50_ values of 97.92 ± 8.03 and 53.70 ± 2.40 µg/ml, respectively, compared to RW30 and RW80 and latex. Acarbose, an antidiabetic drug, also showed IC_50_ value against α‐glucosidase at 82.09 ± 17.099 µg/ml (Mopuri et al., 2018), while the antihypertensive drug captopril exhibited IC_50_ for ACE at 1.3 ng/ml (FitzGerald & Meisel, 2000), suggesting promising properties of REt30 as an antidiabetic compound. Compared to the well‐characterized *F. carica*, its ethanolic fraction of fruit, leaf, and stem bark exhibited IC_50_ values for α‐glucosidase at 255.57, 271.59, and 441.08 µg/ml, respectively, and approximately 2.5–4.5‐fold higher than REt30 (Table 4). Bioactive compound contents suggested that high inhibitory activities, as observed in the ethanolic fraction, might be due to the biological functions of caffeic acid and cyanidin since the contents of both phenolic acids were found to be significantly higher in ethanolic than water fractions (Table 3). Likewise, contents of these phenolic acids were higher in water extracted at higher temperature than lower temperature. Caffeic acid exhibited an IC_50_ value of 149.17 μg/ml against α‐glucosidase and 77.46 μg/ml against ACE (Bhullar et al., 2014; Ishikawa et al., 2007), while cyanidin displayed an IC_50_ of 1.15 μg/ml for α‐glucosidase (Tadera et al., 2006). Interestingly, even though latex exhibited high content of caffeic acid, its activity against α‐glucosidase was not observed. Thus, cyanidin, which was absent in latex, might be the main phenolic acid responsible for anti‐α‐glucosidase activity. This hypothesis was confirmed by higher α‐glucosidase inhibitory activity detected in the fraction containing cyanidin than caffeic acid (Ishikawa et al., 2007; Tadera et al., 2006).

**TABLE 4:** The IC_50_ values and percent inhibition of RW30, RW80, REt30, and latex against *α*‐glucosidase, AChE, ACE and lipase

Sample	IC_50_ (µg/ml)			% Inhibition^a^
	α‐Glucosidase	AChE	ACE	Lipase
RW30	303.05 ± 15.20^A^	115.65 ± 6.15^D^	100.81 ± 7.34^C^	12.02 ± 0.57^C^
RW80	137.95 ± 16.19^B^	874.30 ± 32.81^A^	152.60 ± 8.06^B^	20.61 ± 1.29^B^
REt30	97.92 ± 8.03^C^	452.80 ± 36.49^B^	53.70 ± 2.40^D^	ND
Latex	ND	376.50 ± 18.24^C^	315.20 ± 3.96^A^	39.87 ± 2.87^A^

Note

Data are presented as mean ± *SD* of triplicate experiments. Capital letter within a column for a given parameter are significantly different from each other at *p* <.05. The SPSS program was used to calculate for statistical difference by one‐way analysis of variance (ANOVA) and Duncan's multiple comparison test.

Abbreviation: ND, not detected.

^a^ Final concentration of FD extracts in antilipase assay was 400 µg/ml.

The AChE and lipase inhibitory activities of all FD extracts were low compared to α‐glucosidase and ACE. Even though ethanol could extract more phenolics from FD material than water, these phenolics might not be suitable inhibitors for AChE. Considering the active site of AChE (Bajda et al., 2013), only small and less bulky compounds can fit in, leading to particular types of AChE inhibitors. Interestingly, RW30 extracted using water at low temperature (30°C) exhibited higher AChE inhibitory effectiveness than extraction with water at high temperature (80°C). Thus, data suggested that bioactive compounds extracted from FD root with water might be small compounds that acted as more effective AChE inhibitors than larger compounds from ethanolic extract. Surprisingly, no inhibitory activity was detected in REt30 against lipase, even though this fraction exhibited higher TPCs and TFCs than water extracts (Table 2). Therefore, phenolics might not be the main compounds responsible for antilipase activity in FD root extracts. Previous literature suggested that particular peptides/proteins and polysaccharides play an important role in inhibiting lipase reaction (Gargouri et al., 1986; Hu et al., 2013). Concurring with our data, ethanolic extract of *F. carica* showed comparable low antilipase activity (Mopuri et al., 2018).

### 3.4 Cytotoxicity and cell cycle analysis

**FIGURE 1:**
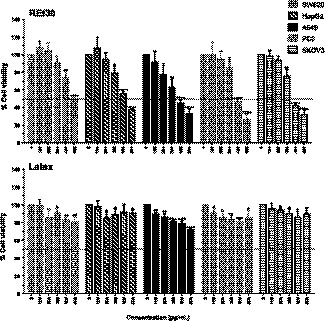
Effect of REt30 and latex on cell viability of human colorectal adenocarcinoma (SW620), human hepatocellular carcinoma (HepG2), human ovarian carcinoma (SKOV‐3), human prostate adenocarcinoma (PC3), and human lung carcinoma (A549). Data are expressed as mean ± standard deviation (*SD*) of three experiments. The percentage of cell viability of each cell line (% cell viability) was calculated, and statistical significance of each cell line was analyzed by Student's unpaired *t* test against its control group. *p <.05; **p <.01; ***p <.001; and ****p <.0001

Several parts of *Ficus* species have been reported for their anticancer activity (Lansky et al., 2008). Here, we examined the cytotoxic effects of FD extracts against five human cancer cell lines as colorectal adenocarcinoma (SW620), hepatocellular carcinoma (HepG2), ovarian carcinoma (SKOV‐3), prostate adenocarcinoma (PC3), and lung carcinoma (A549) treated with various concentrations of up to 200 µg/ml of RW30, REt30, RW80, and latex for 48 hr. Cell percentage viability was investigated by MTT assay. RW30 and RW80 showed no toxicity to all tested cancer cells (Figure S2). REt30 and latex may be toxic to cancer cells. The National Institute of Cancer (NIC) has stipulated that potential anticancer activity from plant extracts should exhibit an IC_50_ value at ≤ 20 μg/ml. REt30 and latex exhibited slight anticancer activity, and the MTT assay was repeated with maximum dose set at 500 µg/ml. Results in Figure 1 and Table 5 show that REt30 caused significantly decreased cancer cell survival, especially for A549, SKOV3, and HepG2 cells with IC_50_ values of 268 ± 46, 323 ± 15, and 337 ± 39 µg/ml, respectively, whereas latex showed minor toxic effects only in A549 cells with an IC_50_ value ≥500 µg/ml. Our data indicated that growth inhibition of cancer cells by REt30 was more effective than latex. Likewise, the selectivity index (SI) of REt30 was assayed in parallel using mouse embryonic fibroblast (3T3‐L1) as a normal cell representative. An SI value ≥2 reflects high selectivity of the extract (Badisa et al., 2006). REt30 exhibited anticancer activity (Table 5), with SI values ranging from 2.3 to 3.0, implying a high degree of selectivity against cancer cells, particularly A549 cells.

**TABLE 5:** The half inhibitory concentration (IC_50_) values and the selectivity index (SI) of REt30 against five cancer cell lines

Cancer cell lines	REt30	
	IC_50_ (µg/ml)	Selectivity Index
SW620	>500	ND
HepG2	337 ± 39	>2.4
A549	268 ± 46	>3.0
PC3	350 ± 11	>2.3
SKOV3	323 ± 15	>2.5
Normal cells (3T3‐L1)	>800	ND

Notes

Data are presented as mean ± *SD* of triplicate experiments. The SI index was calculated as the IC50 ratio of REt30 on 3T3‐L1 to each cancer cell line at the same duration time of treatment.

Abbreviation: ND, not determined.

HPLC analysis (Table 3) showed caffeic acid as the main phenolic compound existing in REt30 at 0.0136 µg/ml in 500 µg/ml extract. Caffeic acid was shown to inhibit A549 growth with an IC_50_ value of 27 µg/ml (Lin et al., 2012), while cyanidin presented in REt30 of 500 µg/ml extract at 0.0023 µg/ml, and was not toxic to A549 cells (Chen et al., 2006). Many studies reported that *Ficus* species are likely to kill cancer cells. The latex of *F. carica* L. inhibited human hypopharynx squamous carcinoma FaDu cells with an IC_50_ value of 25 µg/ml (Shin et al., 2017), while aerial roots of *F. elastic* extracted with methanol showed IC_50_ values of 20 µg/ml against human cervix adenocarcinoma (HeLa cells) (Mbosso Teinkela et al., 2018). However, our results suggested that caffeic acid and cyanidin in FD may not contribute to cancer cell toxicity. Triterpenoid in root and β‐sitosterol in latex have been postulated as anticancer compounds in *Ficus* spp. (Lansky et al., 2008).

REt30 affected the growth of some cancer cells, possibly by disturbing cell cycle progression, while *p*53, a frequently mutated tumor suppressor gene found in cancer, contributed to the sensitivity of cancer cells to chemotherapeutic agents. Thus, A549 (wild‐type *p*53) and SKOV3 (*p*53 mutated) were selected for cell cycle study (Mukhopadhyay & Roth, 1997; Ryu et al., 2009). Progression of A549 and SKOV3 after treatment with REt30 at 300–500 µg/ml was compared with untreated and cisplatin‐treated control. The extract at 500 µg/ml inhibited SKOV3 cell growth at the G2/M phase (approximately 30%; F), while by contrast to cisplatin, REt30 at 300 and 400 µg/ml significantly arrested A549 cells in the G1 phase (approximately 70%–80% and 1.3‐fold greater than the untreated control) (Figure 3). Cell distribution of A549 was not changed by REt30 at 500 µg/ml, possibly due to cell death induction as observed under the microscope (data not shown). An aqueous extract of *F. religiosa* was shown to induce G1 phase arrest of human cervical cancer cells (SiHa, wild‐type *p*53 (Lee et al., 2006)) by disturbing G1 phase proteins (Choudhari et al., 2013). Consistent with our data, damaged cancer cells with wild‐type *p*53 status were arrested at G1 before apoptosis induction (Shaw et al., 1992); however, *p*53 mutant cancer cells underwent G2/M arrest (Kastan et al., 1991). A high dose of REt30 was required to inhibit A549 growth and this should be explored for *p*53‐mediated apoptosis in lung cancer.

**FIGURE 2:**
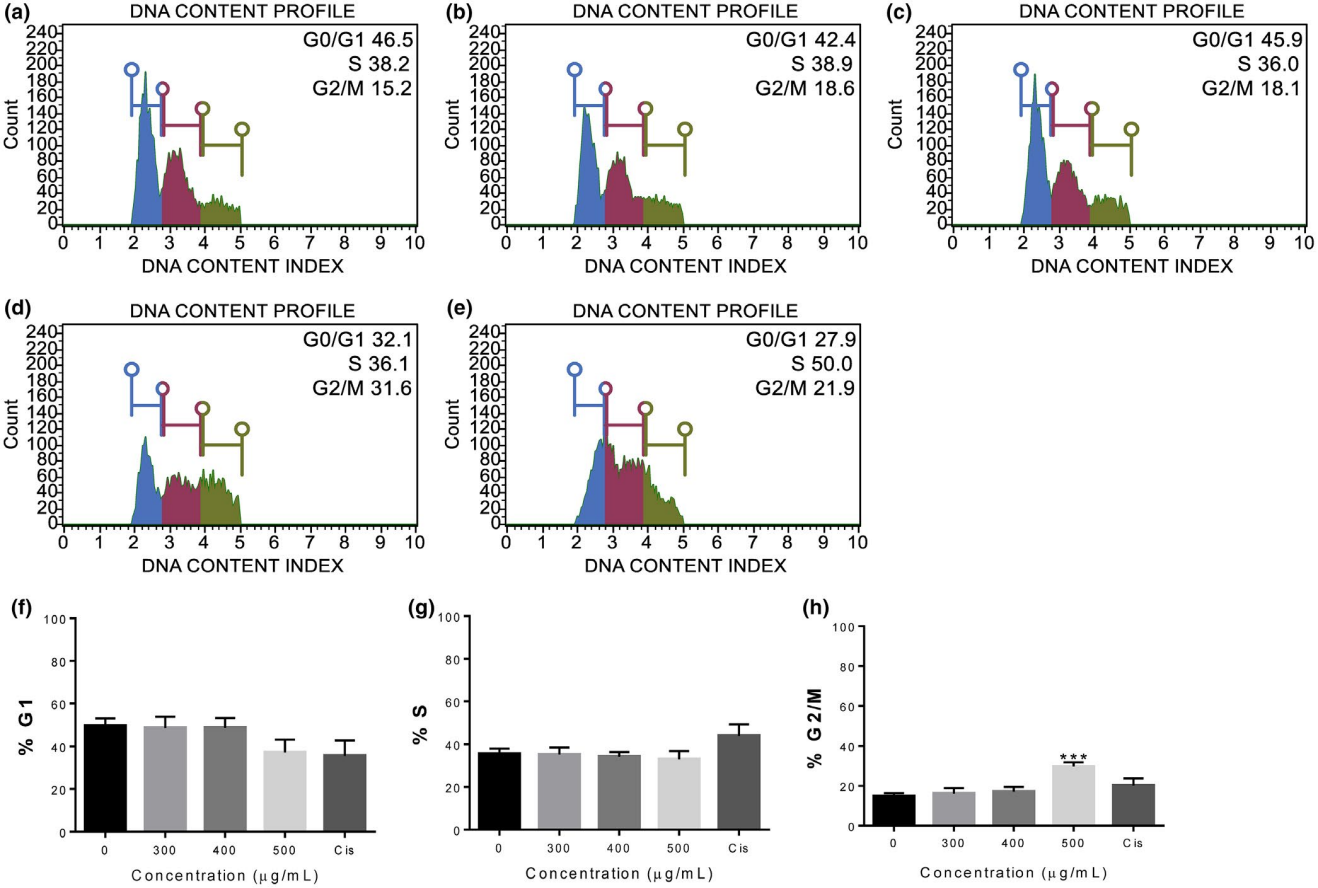
Cell cycle progression of SKOV3 cells treated with REt30 or cisplatin for 48 hr. (a) Untreated, (b) 300 µg/ml of REt30, (c) 400 µg/ml of REt30, (d) 500 µg/ml of REt30, (e) 10 µM of cisplatin, (f) percentage of cells in G1 phase, (g) percentage of cells in S phase, and (h) percentage of cells in G2/M phase. Values are presented as mean ± *SD* of three independent experiments. ***p <.001 vs. control

**FIGURE 3:**
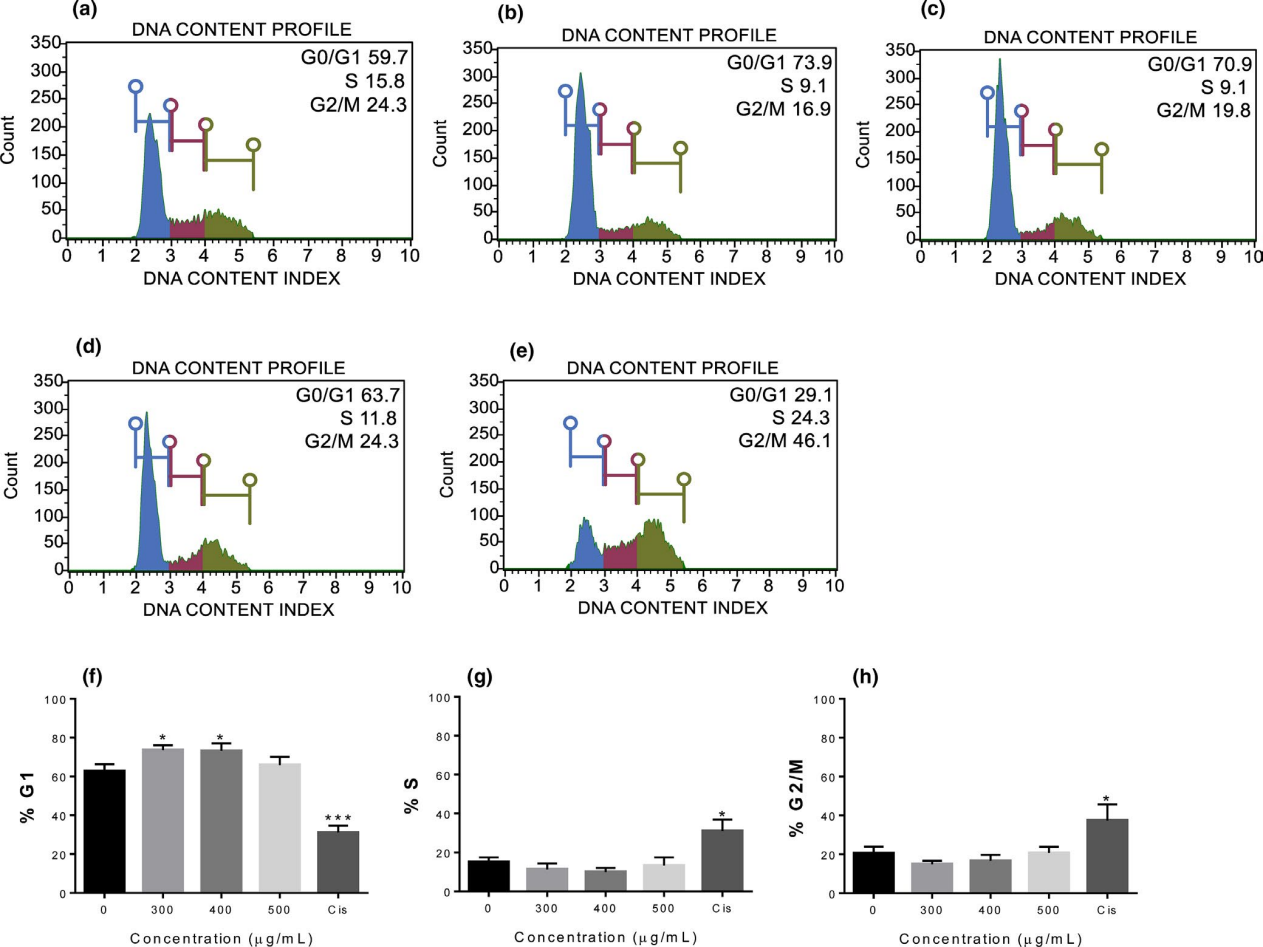
Cell cycle progression of A549 cells treated with REt30 or cisplatin for 48 hr. (a) Untreated, (b) 300 µg/ml of REt30, (c) 400 µg/ml of REt30, (d) 500 µg/ml of REt30, (e) 14 µM of cisplatin, (f) percentage of cells in G1 phase, (g) percentage of cells in S phase, and (h) percentage of cells in G2/M phase. Values are presented as mean ± *SD* of three independent experiments. *p <.05 and ***p <.001 vs. control

### 3.5 Genotoxicity analysis in *Drosophila*

**FIGURE 4:**
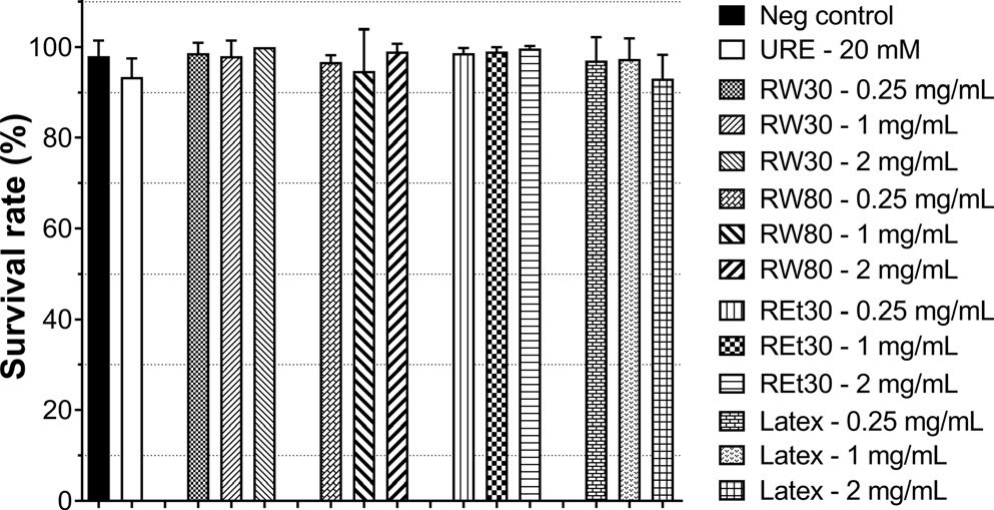
Survival percentage of adult flies obtained from trans‐heterozygous *mwh+/+flr* ^3^ larvae with improved high bioactivation cross‐fed on standard medium (NDM) and experimental medium containing 20 mM urethane or RW30, RW80, REt30, and latex (0.25, 1, and 2 mg/ml)

Scant information regarding genotoxicity has been documented; however, the historical use of *Ficus* species in folk medicine with no reports of any adverse side effects suggests safety in humans. Before investigating FD extract in both animal and human studies, genotoxicity testing is mandatory. An in vivo short‐term assay involving the *Drosophila* wing spot test (SMART) was used to determine the mutagenic properties of FD extract. The SMART assay is a more sensitive mutagenic assay compared to rodents (Delgado‐Rodriguez et al., 1999) and is capable of detecting several types of DNA damages, including point mutations and DNA breaks (Wurgler & Graf, 1985). First, the survival rate of trans‐heterozygous third instar larvae was studied after exposure to FD extracts. The number of surviving flies obtained from larvae raised on each experimental medium containing three different concentrations of RW30, RW80, REt30, and latex is illustrated in Figure 4. Compared to the negative control, results implied that not all FD extracts induced toxicity; therefore, 2 mg/ml was further used for wing spot analysis. Findings in Table 6 show that, in agreement with a previous study (Srichamnong et al., 2018), a nontoxic dose of urethane strongly displayed total spots/wing at 24.63 compared to 0.53 for the negative control. In addition, compared to urethane, RW30, RW80, REt30, and latex revealed no induction of either small, large, or twin spots even at a high dose of 2 mg/ml (Table 6), suggesting that RW30, RW80, REt30, and latex could be considered as genome safe.

**TABLE 6:** Summary of somatic mutation and recombination test data obtained from trans‐heterozygous flies after exposure to RW30, RW80, REt30, or latex at 2 mg/ml

Treatment	Spots/wing (number of spots from 40 wings)			
Sample	Small single (*m* = 2)	Large single (*m* = 5)	Twin spot (*m* = 5)	Total spot (*m* = 2)
Negative control	0.48 (19)	0.05 (2)	0 (0)	0.53 (21)
Urethane, 20mM	15.53 (621)+	7.03 (281)+	2.08 (83)+	24.63 (985)+
RW30 (1st experiment)	0.30 (12)−	0 (0)i	0.03 (1)i	0.33 (13)−
RW30 (2nd experiment	0.23 (9)−	0.08 (3)i	0 (0)i	0.30 (12)−
RW80 (1st experiment)	0.40 (16)−	0 (0)i	0.03 (1)i	0.43 (17)−
RW80 (2nd experiment	0.08 (3)−	0.23 (9)+	0.08 (3)i	0.38 (15)i
REt30 (1st experiment)	0.25 (10)−	0 (0)i	0 (0)i	0.25 (10)−
REt30 (2nd experiment	0.25 (10)−	0.03 (1)i	0 (0)i	0.28 (11)−
Latex (1st experiment)	0.28 (11)−	0 (0)i	0 (0)i	0.28 (11)−
Latex (2nd experiment	0.30 (12)−	0.10 (4)i	0.03 (1)i	0.43 (17)i

^a^ Statistical diagnoses using estimation of spot frequencies and confidence limits according to for comparison with deionized water (Frei & Wurgler, 1988) (negative control); + = positive; − = negative; i = inconclusive. Probability levels: *α* = *β* = 0.05. One‐sided statistical test “*m*” is an increased mutation frequency compared with the spontaneous frequency (*m* times).

We also studied the antigenotoxicity of FD extracts against urethane using the same assay. Before wing analysis, the larvae were cotreated with 20 mM urethane and three concentrations of FD extracts. Again, urethane exhibited total spots/wing at about 30 (Table 7); however, all FD extracts revealed negligible or weak inhibitory effects toward urethane mutagenicity (5%–40% of inhibition). Further investigations using other reference mutagens might elucidate the antimutagenicity potential of FD extract.

**TABLE 7:** Summary of somatic mutation recombination test data and percent inhibition of trans‐heterozygous flies coexposed to 20 mM urethane and RW30, RW80, REt30, and latex at different concentrations

Treatment	Spots per wing (number of spots from 40 wings)				
Sample	Small single spot (*m* = 2)	Large single spot (*m* = 5)	Twin spot (*m* = 5)	Total spot (*m* = 2)	Inhibition (%)^a^
Negative control	0.95 (38)	0 (0)	0.03 (1)	0.98 (39)	—
Urethane (URE), 20mM	18.95 (758)	9.50 (380)	2.15 (86)	30.60 (1,224)	—
RW30 (0.25 mg/ml) + URE	12.33 (493)	7.30 (292)	2.20 (88)	21.83 (873)	29 (W)
RW30 (1 mg/ml) + URE	14.40 (576)	6.10 (244)	2.0 (80)	22.5 (900)	26 (W)
RW30 (2 mg/ml) + URE	12.33 (493)	6.58 (263)	1.75 (70)	20.65 (826)	33 (W)
RW80 (0.25 mg/ml) + URE	13.48 (539)	7.35 (294)	2.28 (91)	23.1 (924)	25 (W)
RW80 (1 mg/ml) + URE	15.70 (628)	5.33 (213)	1.70 (68)	22.73 (909)	26 (W)
RW80 (2 mg/ml) + URE	11.78 (471)	5.13 (205)	1.53 (61)	18.43 (737)	40 (W)
REt30 (0.25 mg/ml) + URE	18.30 (732)	8.18 (327)	2.50 (100)	28.98 (1,159)	5 (N)
REt30 (1 mg/ml) + URE	15.75 (630)	6.65 (266)	1.70 (68)	24.10 (964)	21 (W)
REt30 (2 mg/ml) + URE	16.53 (661)	9.40 (376)	2.35 (94)	28.28 (1,131)	8 (N)
Latex (0.25 mg/ml) + URE	16.28 (651)	7.75(310)	2.33 (93)	26.55 (1,054)	14 (N)
Latex (1 mg/ml) + URE	18.70 (748)	6.90 (276)	1.90 (76)	27.50 (1,100)	10 (N)
Latex (2 mg/ml) + URE	15.35 (614)	5.68 (227)	1.40 (56)	22.43 (897)	27 (W)

^a^ Percent of inhibition = ((A − B)/A) × 100. Where A is the number of total spots per wing of positive urethane control group, B is the number of total spots per wing of each experimental group. “N” Express as negligible effect and “W” express as weak inhibitory effect of 20 mM urethane.

## 4 CONCLUSION

This is the first comprehensive bioactivity and genotoxicity assessment of *F. dubia* root and latex. Our data showed that all FD extracts lacked genotoxicity in vivo. The FD ethanolic root extract (REt30) possessed health‐promoting properties, predominantly anti‐α‐glucosidase, as a possible future novel antidiabetic drug. The extract also specifically exhibited cell growth inhibitory effect against A549 and SKOV3 cancer cells compared to cisplatin as the current drug of choice. The FD extract shows potential for the treatment of diabetes and lung cancer. However, further investigation and identification of its constituent functional compounds are necessary.

## CONFLICT OF INTEREST

All authors declare that there are no conflicts of interest.

## ETHICAL APPROVAL

The *Drosophila* study was approved by Mahidol University‐Institute Animal Care and Use Committee (MU‐IACUC) (COA. No.MU‐IACUC 2018/018).

## Supporting information

Fig S1‐2

## Data Availability

Data available on request from the authors.
