# Erratum

**DOI:** 10.1002/fsn3.2325

**Published:** 2021-05-13

In the article by Suttisansanee et al., (2021), the eighth author name was incorrectly provided. The correct name is “Bhanumas Chantarasuwan.” We apologize for this error.
