# Supplementary material for: Health‐promoting bioactivity and in vivo genotoxicity evaluation of a hemiepiphyte fig, Ficus dubia
Source: Food Sci Nutr. 2021 Mar 3;9(4):2269–79. doi: 10.1002/fsn3.2205 (PMC8020917; doi:10.1002/fsn3.2205)

Supplementary materials:

Health-Promoting Bioactivity and *In vivo* Genotoxicity Evaluation of a Hemiepiphyte Fig, *Ficus dubia*

Uthaiwan Suttisansanee[1,2], Pornsiri Pitchakarn[3], Pisamai Ting[1], Woorawee Inthachat[1,2], Parunya Thiyajai[1], Daraphan Rodthayoy[3], Jirarat Karinchai[3], Bhanumas Chanatarasuwan[4], Onanong Nuchuchua[5], and Piya Temviriyanukul[1,2,*]

[1] Institute of Nutrition, Mahidol University, Salaya, Phuttamonthon, Nakhon Pathom Thailand, 73170

[2]Food and Nutrition Academic and Research Cluster, Institute of Nutrition, Mahidol University, Salaya, Phuttamonthon, Nakhon Pathom 73170, Thailand

[3] Department of Biochemistry, Faculty of Medicine, Chiang Mai University, Meung, Chiang Mai, Thailand, 50200

[4] Thailand Natural History Museum, National Science Museum, Klong Luang, Pathum Thani, Thailand, 12120

[5] National Nanotechnology Center (NANOTEC), National Science and Technology Development Agency (NSTDA), Klong Luang, Pathum Thani, 12120, Thailand

*To whom correspondence should be addressed at Institute of Nutrition, Mahidol University, Salaya, Phuttamonthon, Nakhon Pathom Thailand, 73170. Tel.: +662-800-2380 ext. 116; Fax: +662-441-9344. E-mail: P. Temviriyanukul (piya.tem@mahidol.ac.th).

Supplementary Figure 1.

The HPLC chromatogram of RW30, RW80, REt30 and latex after acid hydrolysis.

Sample	Chromatogram		
	**280 nm**	**338 nm**	**524 nm**
RW30, 50 mg/mL	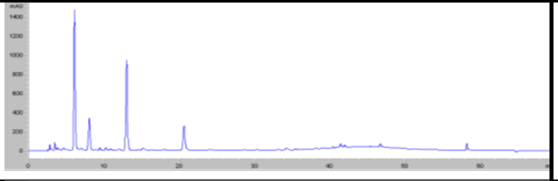	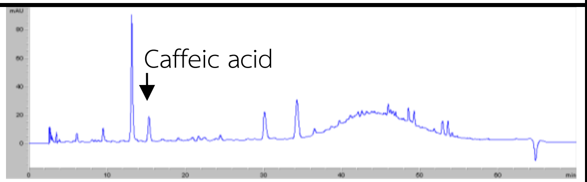	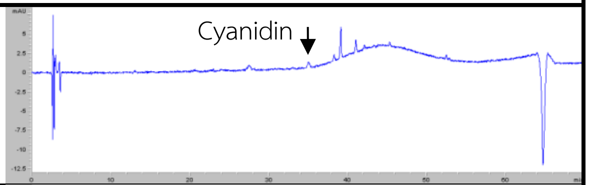
RW80, 50 mg/mL	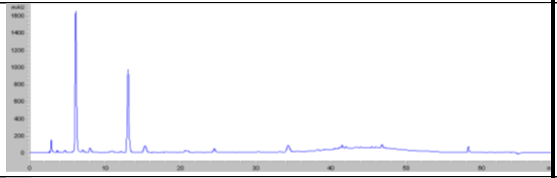	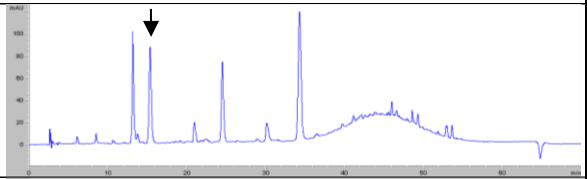	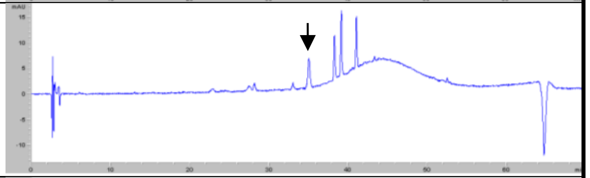
REt30, 50 mg/mL	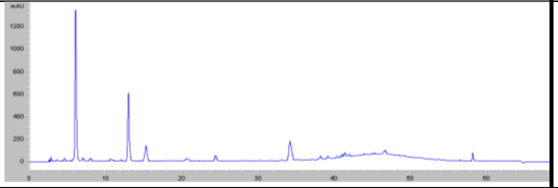	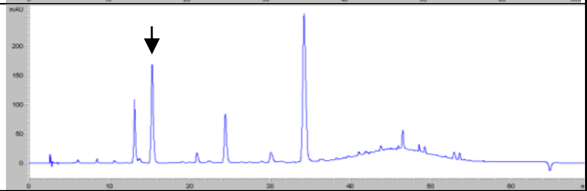	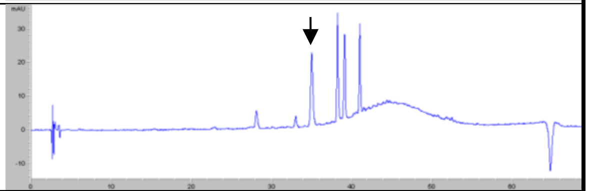
Latex, 20 mg/mL	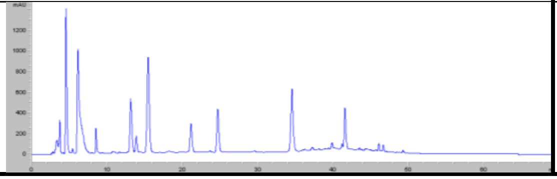	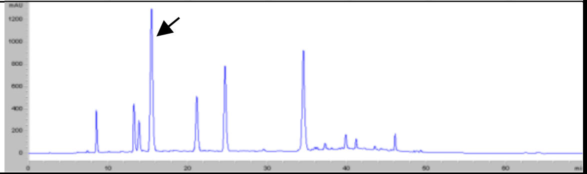	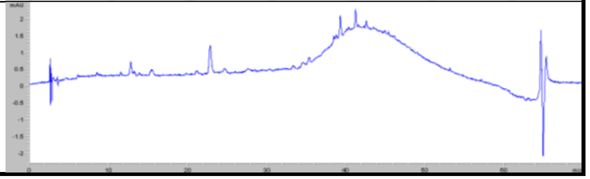

Supplementary Figure 2.

Effect of FD extracts (0-200 µg/mL) on cell viability of human colorectal adenocarcinoma (SW620), human hepatocellular carcinoma (HepG2), human ovarian carcinoma (SKOV-3), human prostate adenocarcinoma (PC3) and human lung carcinoma (A549). Data are expressed as mean ± standard deviation (SD) of three experiments. Percentage of cell viability of each cell line (% cell viability) was calculated compared to the control group.

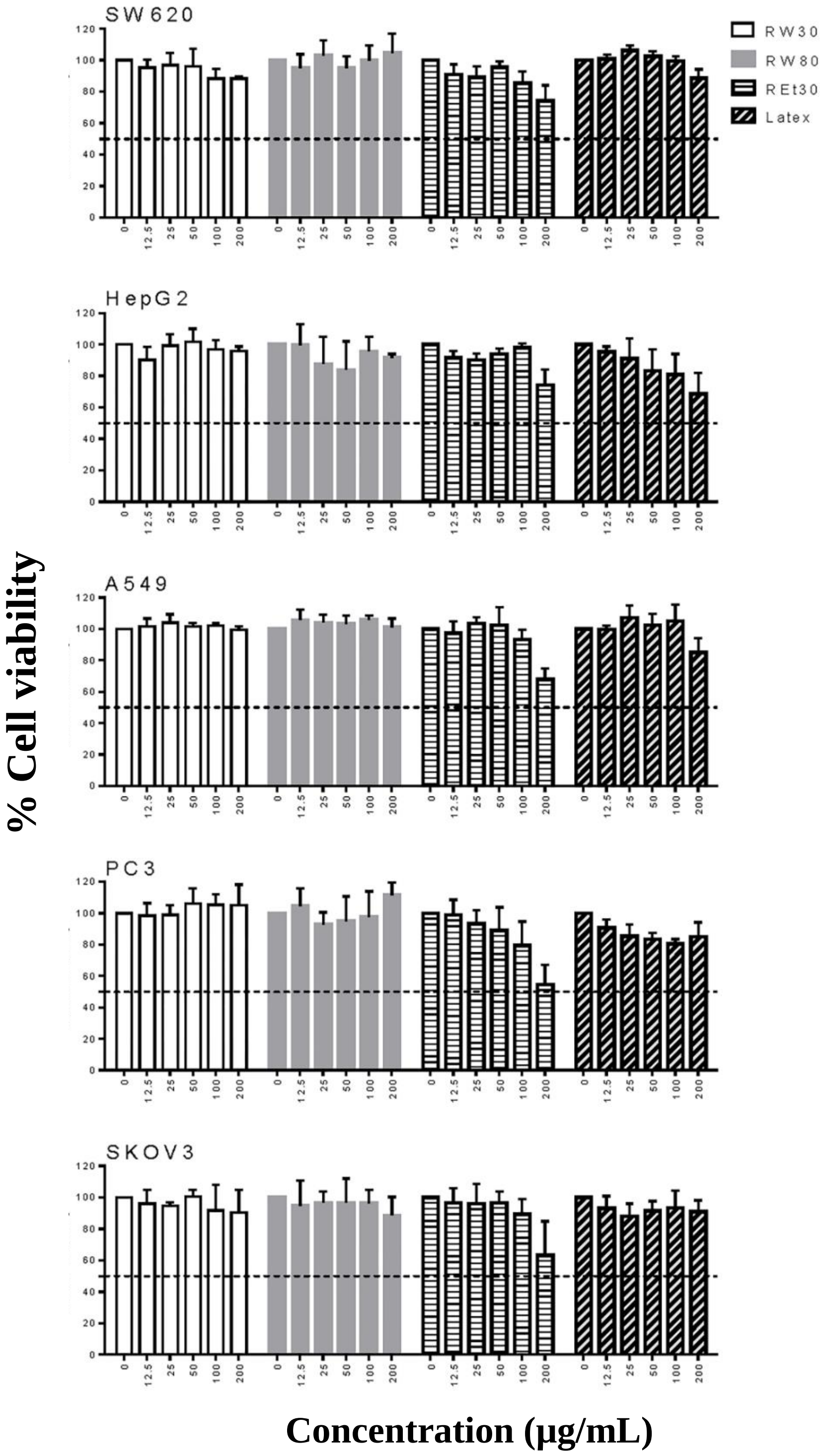

Supplement: Supplementary file 1 — Fig S1‐2 [file FSN3-9-2269-s001.pdf]
